# Adding checkpoint inhibitors to first-line chemotherapy for NUT carcinoma patients

**DOI:** 10.1038/s41698-024-00768-7

**Published:** 2025-01-25

**Authors:** Sarah Haebe, Gesa Schuebbe, Philipp Jurmeister, Michael von Bergwelt-Baildon, C. Benedikt Westphalen, Ulrich M. Lauer, Wolfgang G. Kunz, Marion Subklewe, Oliver Weigert

**Affiliations:** 1 Department of Medicine III, LMU University Hospital, Munich, Germany; 2 Department of Pathology, LMU University Hospital, Munich, Germany; 3 German Cancer Consortium (DKTK), Partner Site Munich, Munich, Germany; 4 Comprehensive Cancer Center (CCC Munich LMU), LMU University Hospital, Munich, Germany; 5 Department of Medicine VIII, University Hospital Tübingen, Tübingen, Germany; 6 Department of Radiology, LMU University Hospital, Munich, Germany

**Keywords:** Genetics, Cancer, Cancer immunotherapy

## Abstract

Rare cancers present significant challenges in diagnosis, treatment, and research, accounting for up to 25% of global cancer cases. Due to their rarity and atypical presentations, they are often misdiagnosed, resulting in late-stage detection and poor outcomes. Here, we describe a patient case with advanced metastatic nasopharynx NUT carcinoma, one of the rarest and most aggressive cancers. We conducted a comprehensive analysis of this patient’s tumor, including Tumor Mutation Burden, Microsatellite Instability, and genetic profiling to explore further putative druggable targets. The tumor exhibited high PD-L1 expression and showed a notable response to immune checkpoint inhibitors when combined with platinum-based radio-chemotherapy. Our findings indicate that checkpoint inhibitors could play a critical role in treating NUT carcinoma, offering new therapeutic avenues and hope for patients with this challenging diagnosis. Whether PD-L1 expression may be a useful predictor of immune checkpoint inhibitor efficacy warrants further research.

## Introduction

Rare cancers pose unique challenges in diagnosis, treatment, and research, accounting for up to 25% of all cancers diagnosed worldwide^1^. One of the primary challenges faced by patients with rare cancers is the difficulty in obtaining timely and accurate diagnoses. Due to their infrequency and often atypical presentation, rare cancers are frequently misdiagnosed or diagnosed only at advanced stages, leading to poorer outcomes. The lack of awareness among healthcare providers about these rare entities contributes further to delays in diagnosis and appropriate treatment initiation. Additionally, scarce clinical expertise and a lack of standard therapy can result in suboptimal treatment decisions and worse outcomes. Limited research interest and funding exacerbate these challenges, resulting in insufficient data on pathogenesis, prevalence, and optimal treatment strategies^1–3^.

Yet, well-documented and thoroughly analyzed individual case reports can be highly informative. Moreover, the therapeutic success of immune checkpoint inhibitors (ICIs) across multiple entities is a promising avenue also for rare cancers.

Here, we present a clinical case of one of the rarest and most aggressive cancers worldwide^4^, NUT carcinoma. NUT carcinoma represents a highly aggressive subtype of squamous cell carcinoma. The actual incidence is still unknown; only about 300 cases have been reported in the literature to date^4^. It predominantly originates from midline structures (thoracic, head, and neck) and affects patients of all ages, though it is most observed in adolescents and young adults^5^. NUT carcinoma is molecularly characterized by an aberrant *NUTM1* fusion gene on chromosome 15^6^. In about 70% of cases, *NUTM1* is fused to BRD4, followed by BRD3, NSD3, ZNF532, ZNF592, and CIC^7–9^. Typically diagnosed at an advanced stage, the median overall survival (OS) is merely 6.5 months^5,10^. Given its rarity, there is no standard-of-care therapy for advanced-stage NUT carcinoma. Platinum-based chemotherapies are commonly used, often combined with other agents or radiotherapy, but with limited success^11–13^. In our case, we observed an intriguing response to ICI when added to a platinum-based radio-chemotherapy. Moreover, we provide a comprehensive view of the tumor characteristics, including Tumor Mutation Burden (TMB), Microsatellite Instability (MSI), PD-L1 Tumor Proportion Score (TPS), and genetic profiling.

## Results

### Case presentation

**Fig. 1:**
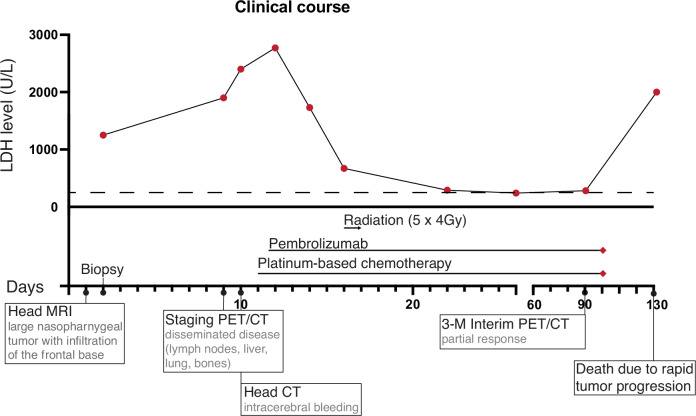
Timeline of the clinical course including the LDH blood levels (top) and the treatment and imagine regime applied during the clinical course as well as days since initial hospital admission (bottom) are indicated. CT computerized tomography, MRI magnetic resonance imaging.

**Fig. 2:**
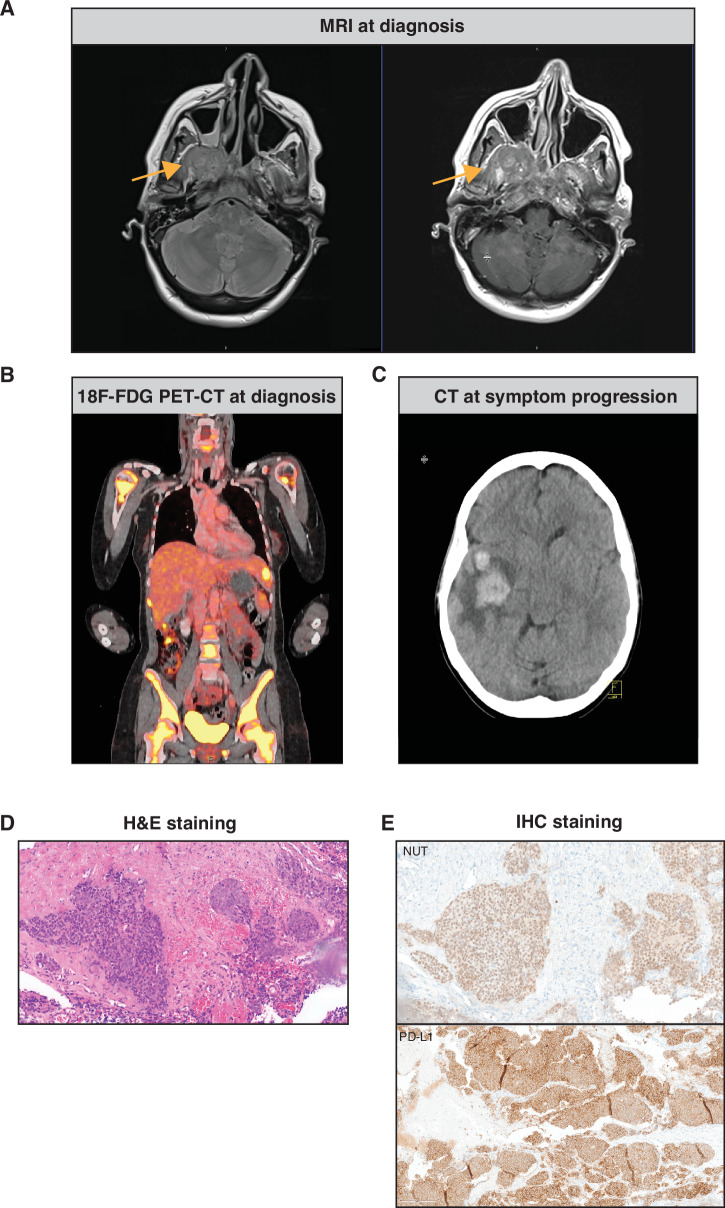
Diagnosis of NUT carcinoma: Imaging and Histology. **A** Magnetic resonance imaging (MRI) scan of the head shows a large right-sided contrast-enhanced tumor of the nasopharynx with infiltration of the frontal base and with encasement of the cavernous sinus, including both internal carotid arteries. **B** Whole body F18-FDG PET-CT scan revealed hypermetabolic metastatic disease, including metastases in the lymph nodes, lungs, liver, and bones. **C** Computerized tomography (CT) scan of the head with contrast demonstrated the nasopharyngeal tumor with new bleeding in the right temporal lobe. **D** Hematoxylin and eosin (H&E) staining revealed nests of round-oval malignant cells with finely dispersed chromatin and prominent eosinophilic nucleoli. **E** Immunohistochemistry (IHC) staining of the nasopharyngeal tumor with NUT antibody (top) and with PD-L1 antibody (bottom).

A 41-year-old woman was referred to our clinic with a 3-week history of increasing right-sided facial sensory disturbances accompanied by escalating neuralgic pain, headache, and nausea. MRI scan of the head revealed a nasopharyngeal mass (5 cm in diameter) with infiltration of the frontal base, encasement of the cavernous sinus as well as of both internal carotid arteries (Figs. 1 and 2A). Moreover, the scan exhibited an extension of the tumor into the right temporal fossa and posterior maxillary sinus and an intra-axial infiltration into the right temporo-basal region, accompanied by surrounding edema. Blood test showed an LDH level of 1200 U/l (reference ≤250 U/L, Fig. 1). Whole body F18-FDG PET-CT scan revealed widespread metastatic disease, including metastases in the lymph nodes, lungs, liver, and bones (Fig. 2B). Her symptoms rapidly progressed, and a CT scan of the head revealed progression of the edema and an intracranial bleeding into the right temporal lobe (Fig. 2C). Histopathology showed enlarged cells with finely dispersed chromatin and prominent eosinophilic nucleoli, suspicious for squamous cell carcinoma (Fig. 2D). Lacking surgical options, a decision was made to treat this patient conservatively. Because of rapidly increasing LDH levels (2500 U/L), we initiated platinum-based chemotherapy. Concurrently, the patient underwent radiotherapy at the nasopharynx and frontal base with 20 Gray applied in 5 fractions for 4 days.

Given the tumor’s midline location and unusual aggressiveness, we suspected a NUT carcinoma. Indeed, immunohistochemistry (IHC) was positive for the hallmark protein NUT^14^ (Fig. 2E top). IHC exhibited moreover p40, p63, and p16, whereas CK7 and chromogranin A were found to be negative. Of note, PD-L1 TPS was 100% (Fig. 2E bottom), a remarkably high expression level that had not been previously reported in NUT carcinoma patients. Therefore, we decided to incorporate pembrolizumab to our first-line treatment consisting of cisplatin (q3) und paclitaxel (weekly).

After finishing radiotherapy and the first cycle of chemotherapy plus ICI, her symptoms improved, and bleeding ceased. Moreover, there was a notable decrease in LDH levels (Fig. 1), indicating treatment response. LDH levels can serve as a surrogate marker for proliferation and treatment success in NUT carcinoma patients^15,16^. The patient was able to be discharged home in good overall condition (Eastern Cooperative Oncology Group (ECOG) Performance Status 0). Another two cycles of chemotherapy plus ICI were administered in an outpatient setting.

### Molecular profiling of the tumor

Some fusion partners of *NUTM1* seem to have a prognostic impact in NUT carcinoma patients^5^. Therefore, we performed RNA-sequencing combined with Archer FusionPlex to identify the NUTM1 fusion partner. We detected a ZNF592-NUTM1 fusion, which has not yet been linked to outcome^5^. We further conducted an extensive profiling of the tumor’s genetic and transcriptional characteristics by performing TruSight Oncology NGS assays. We assessed the TMB based on a 1.94 Mb territory (2.4 mutations/Mb) and MSI using 125 microsatellite loci (2.61, cutoff: 20). We also did not detect any homologous recombination deficiency (HRD, Genetic Instability Score (GIS): 1 (cut-off: 42)), significant somatic mutations, gene fusions, or amplifications at both DNA and RNA levels. Of note, NUTM1 gene fusions are generally not included in this assay and, therefore, cannot be detected. The patient’s case was reviewed by our precision oncology specialists. Unfortunately, since there were no pathologic mutations detected, personalized, targeted therapy was not available at the initial diagnosis.

### Subsequent clinical course

**Fig. 3:**
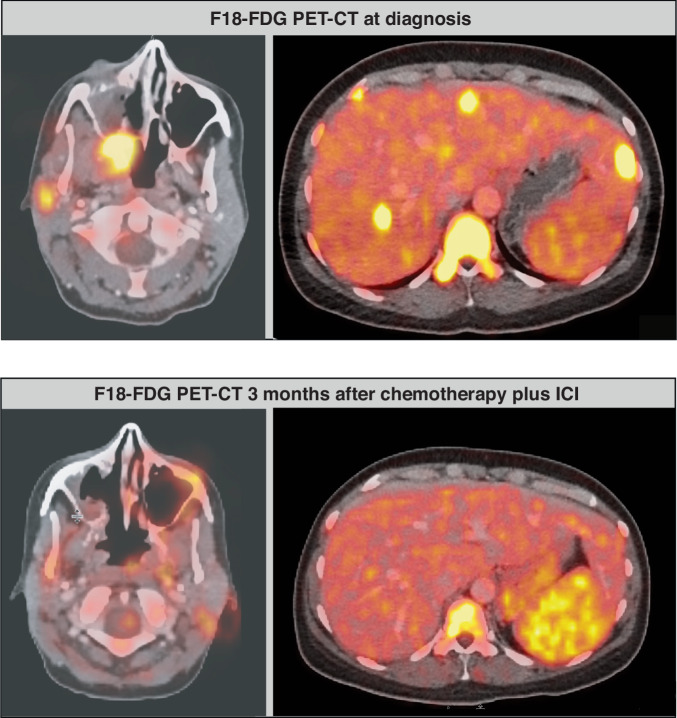
Interim whole-body F18-FDG PET-CT. Whole-body F18-FDG PET-CT staging revealed initial tumor regression and decreased metabolic uptake of the primary nasopharyngeal tumor, as well as of the liver metastasis (bottom), compared to the initial staging at diagnosis (top).

Three months after her initial diagnosis of NUT carcinoma of the nasopharynx with brain infiltration and intracerebral bleeding, the patient was in good general health and experienced only transient mild headache and facial sensory disturbance. Her overall condition was excellent, with an ECOG performance status of 0. The interim whole-body F18-FDG PET-CT scan indicated a partial response (PR) (Fig. 3).

Unfortunately, after 3 cycles of chemotherapy plus ICI, she experienced grade 3 hematologic toxicity (thrombocytopenia) and ICI-related toxicity, including thyroiditis and pneumonitis. ICI treatment had to be stopped, and continuation of treatment was not feasible. Five weeks after discontinuation, a CT scan revealed a massive progression of liver metastasis and tumor-infiltrated lymph nodes. This coincided with LDH levels rapidly increasing again to over 2000 U/L (Fig. 1). The patient died 4 months after her initial diagnosis of NUT carcinoma.

## Discussion

NUT carcinoma is among the rarest cancers but is increasingly recognized as broader tumor genome sequencing approaches become more widely utilized^16^. There is no current effective standard therapy. Treatment, if possible, involves a combination of surgery, platin-based chemotherapy, and radiotherapy. However, outcomes with aggressive multimodal treatment approaches remain poor^11–13^. Thus, there is an unmet need to develop new therapeutic strategies, including immunotherapy and personalized targeted therapy.

Given the discovery of oncogenic fusion drivers, NUT carcinoma has become a promising candidate for targeted therapy. BRD4 and BRD3 are part of the BET protein family. ZNF592 and ZNF532 are zinc finger proteins, identified as a component of the BRD4-NUT complex, crucial for the growth and/or viability of NUT carcinomas^17^. Targeted therapy, such as histone deacetylase inhibitors, to reduce the hyperacetylated chromatin in the BRD4 mega-domains, and BET inhibitors are under clinical investigation, with reported treatment responses in case-based evidence^16,18^. Additionally, other available targeted drugs may be effective in this disease, such as anlotinib, a novel multi-targeting tyrosine kinase inhibitor, that has shown promising outcomes in 2 patients with NUT carcinoma^19,20^. However, the response of targeted therapy is not sustainable, necessitating rational approaches for combination therapies. For instance, combining CDK4/6 inhibitors or EZH2 inhibitors with BET inhibitors has demonstrated synergy, and currently, new phase I combination trials are being tested in NUT carcinoma in the USA (NCT05372640, NCT05019716, NCT05488548).

Besides molecular targeted therapies, ICIs represent another emerging approach to improve treatment response in tumors. While a recent study reported that ICI did not have an impact on progression-free survival or OS when analyzing all systemic therapies, it did show a trend towards improved OS when applied in a first-line setting^16^. The reported median TPS was 0% (range 0–70%), and one case with a TPS of 70% had no response to atezolizumab. In contrast, our patient had a TPS of 100%, and received radio-chemotherapy plus ICI, with was well tolerated and resulted for the first time in marked clinical improvement and a PR in a patient diagnosed with nasopharyngeal NUT carcinoma. Moreover, recent studies demonstrated disease control in both primary and refractory/relapsed patients with pulmonary NUT carcinoma when treated with chemotherapy plus ICIs (Supplementary Table 1)^11,21–23^. Multiple treatment mechanisms, including ICI, targeted therapies, or local immunogenic agents such as intratumoral application of oncolytic virotherapy^16^ may be necessary to combat such an aggressive tumor. Interestingly, in mouse models and various human cell lines, BET inhibitors and ICIs resulted in synergistic therapeutic effects^24,25^.

Comprehensive molecular profiling has significantly advanced our understanding of the molecular landscape of rare cancers. Despite remarkable progress, molecular targeting therapies have, however, failed to yield substantial clinical benefits for patients with NUT carcinoma.

Here, we present a compelling patient case highlighting the potential efficacy of ICI when combined with radiation and chemotherapy in the frontline setting. We observed remarkable initial response to this combination treatment, but also rapid progression upon discontinuation of ICI, suggesting that the addition of ICI contributed to the strong initial treatment response. Whether PD-L1 expression can serve as predictor to ICI remains to be determined, warranting future research and clinical validation.

## Methods

### Patient consent

The patient signed informed consent regarding molecular analyses and publication of her data and imaging studies. All studies were approved by the Ludwig-Maximilian-University Ethics Committee (#3458) and conducted in compliance with German legislation and the Declaration of Helsinki.

### PD-L1 assessment

Immunohistochemistry staining for PD-L1 was conducted using the Ventana SP263 assay (Roche Diagnostics), as previously described^26^.

### Targeted RNA-sequencing

Paraffin-embedded slides were microdissected to obtain >70% neoplastic cells. Neoplastic cellularity was estimated from the sequential slides, which highly reflect the cellularity of the section used for RNA sequencing. Total RNA was isolated with RNAeasy Kit (Qiagen). Libraries were prepared using the Archer FusionPlex Lung v2 (Integrated DNA Technologies) for Illumina sequencing according to the manufacturer’s instructions.

### TruSight Oncology 500 assays

Paraffin-embedded slides were microdissected to obtain more than 70% neoplastic cells. DNA was isolated using the GeneRead DNA FFPE Kit (Qiagen), and RNA was isolated using the RNeasy Kit (Qiagen). Hybridization-capture-based Next-Generation Sequencing was performed using the TruSight Oncology 500 and TruSight Oncology 500 HRD assays (Illumina).

## Supplementary information

Supplementary Table 1

## Data Availability

The data of this study may be available on reasonable request to the corresponding author.

## References

[1] de Heus, E. et al. Unmet supportive care needs of patients with rare cancer: a systematic review. *Eur. J. Cancer Care* **30**, e13502 (2021). 10.1111/ecc.13502 34409667

[2] Pillai, R. K. & Jayasree, K. Rare cancers: challenges & issues. *Indian J. Med. Res.* **145**, 17–27 (2017). 28574010 10.4103/ijmr.IJMR_915_14 PMC5460568

[3] Gatta, G. et al. Rare cancers are not so rare: the rare cancer burden in Europe. *Eur. J. Cancer* **47**, 2493–2511 (2011). 22033323 10.1016/j.ejca.2011.08.008

[4] Lee, T. et al. Prevalence of NUT carcinoma in head and neck: Analysis of 362 cases with literature review. *Head Neck* **42**, 924–938 (2020). 31903701 10.1002/hed.26067

[5] Chau, N. G. et al. An anatomical site and genetic-based prognostic model for patients with nuclear protein in testis (NUT) midline carcinoma: analysis of 124 patients. *JNCI Cancer Spectr.* **4**, pkz094 (2020). 32328562 10.1093/jncics/pkz094 PMC7165803

[6] French, C. A. NUT midline carcinoma. *Cancer Genet. Cytogenet.* **203**, 16–20 (2010). 20951314 10.1016/j.cancergencyto.2010.06.007 PMC3000636

[7] French, C. A. et al. NSD3-NUT fusion oncoprotein in NUT midline carcinoma: implications for a novel oncogenic mechanism. *Cancer Discov.* **4**, 928–941 (2014). 24875858 10.1158/2159-8290.CD-14-0014 PMC4125436

[8] French, C. A. et al. BRD-NUT oncoproteins: a family of closely related nuclear proteins that block epithelial differentiation and maintain the growth of carcinoma cells. *Oncogene* **27**, 2237–2242 (2008). 17934517 10.1038/sj.onc.1210852

[9] Alekseyenko, A. A. et al. Ectopic protein interactions within BRD4-chromatin complexes drive oncogenic megadomain formation in NUT midline carcinoma. *Proc. Natl Acad. Sci. USA* **114**, E4184–e4192 (2017). 28484033 10.1073/pnas.1702086114 PMC5448232

[10] Bauer, D. E. et al. Clinicopathologic features and long-term outcomes of NUT midline carcinoma. *Clin. Cancer Res.* **18**, 5773–5779 (2012). 22896655 10.1158/1078-0432.CCR-12-1153 PMC3473162

[11] Xie, X. H. et al. Clinical features, treatment, and survival outcome of primary pulmonary NUT midline carcinoma. *Orphanet J. Rare Dis.* **15**, 183 (2020). 32650830 10.1186/s13023-020-01449-x PMC7350189

[12] Zhao, R. et al. NUT carcinoma of the lung: a case report and literature analysis. *Front. Oncol.* **12**, 890338 (2022). 35903693 10.3389/fonc.2022.890338 PMC9321640

[13] Joel, S., Weschenfelder, F., Schleussner, E., Hofmann, G. O. & Weschenfelder, W. NUT midline carcinoma in a young pregnant female: a case report. *World J. Surg. Oncol.* **18**, 290 (2020). 33160369 10.1186/s12957-020-02065-6 PMC7648955

[14] Haack, H. et al. Diagnosis of NUT midline carcinoma using a NUT-specific monoclonal antibody. *Am. J. Surg. Pathol.* **33**, 984–991 (2009). 19363441 10.1097/PAS.0b013e318198d666 PMC2783402

[15] Kloker, L. D. et al. Case report: Immunovirotherapy as a novel add-on treatment in a patient with thoracic NUT carcinoma. *Front. Oncol.* **12**, 995744 (2022). 36387105 10.3389/fonc.2022.995744 PMC9647065

[16] Kloker, L. D. et al. Clinical management of NUT carcinoma (NC) in Germany: analysis of survival, therapy response, tumor markers and tumor genome sequencing in 35 adult patients. *Lung Cancer* **189**, 107496 (2024). 38301600 10.1016/j.lungcan.2024.107496

[17] Shiota, H. et al. “Z4” complex member fusions in NUT carcinoma: implications for a novel oncogenic mechanism. *Mol. Cancer Res.* **16**, 1826–1833 (2018). 30139738 10.1158/1541-7786.MCR-18-0474 PMC6279489

[18] Cheng, M. L. et al. Exceptional response to bromodomain and extraterminal domain inhibitor therapy with BMS-986158 in BRD4-NUTM1 NUT carcinoma harboring a BRD4 splice site mutation. *JCO Precis. Oncol.* **7**, e2200633 (2023). 37384867 10.1200/PO.22.00633 PMC10581614

[19] Chai, P., Zhou, C., Jia, R. & Wang, Y. Orbital involvement by NUT midline carcinoma: new presentation and encouraging outcome managed by radiotherapy combined with tyrosine kinase inhibitor: a case report. *Diagn. Pathol.* **15**, 2 (2020). 31900183 10.1186/s13000-019-0922-1 PMC6942267

[20] Jiang, J., Ren, Y., Xu, C. & Lin, X. NUT midline carcinoma as a primary lung tumor treated with anlotinib combined with palliative radiotherapy: a case report. *Diagn. Pathol.* **17**, 4 (2022). 34996489 10.1186/s13000-021-01188-y PMC8742416

[21] Davis, A., Mahar, A., Wong, K., Barnet, M. & Kao, S. Prolonged disease control on nivolumab for primary pulmonary NUT carcinoma. *Clin. Lung Cancer* **22**, e665–e667 (2021). 33349572 10.1016/j.cllc.2020.10.016

[22] Hung, Y. P. et al. Thoracic nuclear protein in testis (NUT) carcinoma: expanded pathological spectrum with expression of thyroid transcription factor-1 and neuroendocrine markers. *Histopathology* **78**, 896–904 (2021). 33231320 10.1111/his.14306

[23] Riess, J. W. et al. Genomic profiling of solid tumors harboring BRD4-NUT and response to immune checkpoint inhibitors. *Transl. Oncol.* **14**, 101184 (2021). 34333275 10.1016/j.tranon.2021.101184 PMC8340305

[24] Hogg, S. J. et al. BET-bromodomain inhibitors engage the host immune system and regulate expression of the immune checkpoint ligand PD-L1. *Cell Rep.* **18**, 2162–2174 (2017). 28249162 10.1016/j.celrep.2017.02.011 PMC5340981

[25] Zhu, H. et al. BET bromodomain inhibition promotes anti-tumor immunity by suppressing PD-L1 expression. *Cell Rep.* **16**, 2829–2837 (2016). 27626654 10.1016/j.celrep.2016.08.032 PMC5177024

[26] Rebelatto, M. C. et al. Development of a programmed cell death ligand-1 immunohistochemical assay validated for analysis of non-small cell lung cancer and head and neck squamous cell carcinoma. *Diagn. Pathol.* **11**, 95 (2016). 27717372 10.1186/s13000-016-0545-8 PMC5055695

