# Supplementary material for: Adding checkpoint inhibitors to first-line chemotherapy for NUT carcinoma patients
Source: NPJ Precis Oncol. 2025 Jan 25;9:26. doi: 10.1038/s41698-024-00768-7 (PMC11762287; doi:10.1038/s41698-024-00768-7)
Supplement: Supplementary file 1 — Supplementary Table 1 [file 41698_2024_768_MOESM1_ESM.docx]

**Supplementary Table 1. First- and second-line immunotherapy treatments in patients with NUT carcinoma**

| #no. | Age | Sex | Primary Site | Metastasis | NUTM1 fusion gene | PD-L1  TPS | Treatment Line | Treatment | ICI | Tox | Outcome | OS | Ref |
| --- | --- | --- | --- | --- | --- | --- | --- | --- | --- | --- | --- | --- | --- |
| # 1 | 57 | M | Lung | Yes | n/a | n/a | 2^nd^ | ICI | Nivolumab | n/a | PFS-2: 12w | 4m | ^1^ |
| #1  #2  #3 | 23  74  31 | M  M  F | Lung  Lung  Lung | Yes  Yes  No | Not detected  Not detected  CHRM5 | n/a | 2^nd^  2^nd^  2^nd^ | ICI  ICI  Chemo+ICI | Atezolizumab  Pembrolizumab  Nivolumab,  Pembrolizumab | n/a  n/a  n/a | PFS-2: 2w  PFS-2: 5m  PFS-2: 4w | 2m  20m  >12m | ^2^ |
| #1  #2  #3 | 34  45  48 | M  M  F | Lung  Lung  Lung | Yes  No  No | n/a  n/a  n/a | 80  70  1 | 1^st^  1^st^  1^st^ | Chemo+ICI  Chemo+ICI  Chemo+ICI | Pembrolizumab  Pembrolizumab  Pembrolizumab | n/a  n/a  n/a | n/a | n/a  >12m  >12m | ^3^ |
| #1 | 34 | F | Lung | Yes | Not reported | 0 | 2^nd^ | ICI | Pembrolizumab | n/a | PD | >5m | ^4^ |
| #1  #2 | 53  39 | F  F | Lung  Lung | Yes  No | BRD4  BRD4 | 0  1 | 2^nd^  2^nd^ | ICI  Chemo+ICI | Nivolumab  Atezolizumab | n/a | PFS-2: 4m  PFS-2: 5m | >4m  ~8m | ^5^ |
| #1  #2 | 31  53 | M  M | Lung  Lung | No  Yes | NSD3  BRD3 | n/a | 1^st^  1^st^ | RCT+ICI  RCT+ICI | Atezolizumab  Nivolumab | n/a  n/a | PFS: 2m  n/a | 2m  12m | ^6^ |
| #1 | 31 | F | Lung | No | n/a | 10 | 2^nd^ | ICI | Nivolumab | Hypo-thyr. II° | PFS-2: 29m | 5y | ^7^ |
| #1 | 39 | M | Nasopharynx | yes | YAP1 | CPS 25 | 1^st^ | Clinical Trial (ICI+TLR7 agonist) | Not reported | n/a | PFS: 1m | 14m | ^8^ |
| #1 | 47 | M | Intracranial | Yes | BRD4 | n/a | 2^nd^ | RCT+ICI | Pembrolizumab | n/a | PFS-2: 3-4m | 4m | ^9^ |
| #1 | 49 | M | Lung | Yes | BRD4 | n/a | Consoli-dation | ICI | Durvalumab | n/a | PFS: 6m | 16m | ^10^ |
| #1 | 24 | M | Lung | Yes | n/a | 0 | 1^st^ | Chemo+ICI | Pembrolizumab | DM  Type I | PFS: ~3m | >24m | ^11^ |
| N=35  N=10* | 40  (18-67) | M=23 | 19 vs 16**  10 vs 2** | Yes for N=27 | BRD4=13 BRD3=3  NSD3=4 | 0 | First | Surgery, N=15  Chemo, N=15  Radiatio, N=4  Chemo+ICI | Pembrolizumab, Atezolizumab,  Nivolumab,  Ipilimumab | n/a | n/a | 228d  229d | ^12^ |
| #1 | 35 | M | Lung | Yes | BRD4 | n/a | First | RCT+ICI | Durvalumab | n/a | PFS: 3m | 6m | ^13^ |

* 10 of 35 cases were treated with chemotherapy plus immune checkpoint inhibitors

** Thoracic versus non-thoracic

CPS, combined proportion score. F, female. ICI, immune checkpoint inhibitor, M, male. N/a, not applicable. OS, overall survival. PFS, progression free survival after first-line therapy. PFS-2, progression free survival after second-line therapy. PD, progressive disease. RCT, radiochemotherapy. TPS, tumor proportion score.

**Supplementary References**

1. Maruyama, N.*, et al.* Nuclear Protein in Testis Carcinoma of the Thorax. *Intern Med* **57**, 3169-3173 (2018).

2. Xie, X.-H.*, et al.* Clinical features, treatment, and survival outcome of primary pulmon/ary NUT midline carcinoma. *Orphanet Journ/al of Rare Diseases* **15**, 183 (2020).

3. Cho, Y.A.*, et al.* Clinicopathological characteristics of primary lung nuclear protein in testis carcinoma: A single-institute experience of 10 # s. *Thorac Cancer* **11**, 3205-3212 (2020).

4. Joel, S., Weschenfelder, F., Schleussner, E., Hofmann, G.O. & Weschenfelder, W. NUT midline carcinoma in a young pregn/ant female: a #report. *World J Surg Oncol* **18**, 290 (2020).

5. Riess, J.W.*, et al.* Genomic profiling of solid tumors harboring BRD4-NUT and response to immune checkpoint inhibitors. *Transl Oncol* **14**, 101184 (2021).

6. Hung, Y.P.*, et al.* Thoracic nuclear protein in testis (NUT) carcinoma: expanded pathological spectrum with expression of thyroid transcription factor-1 and neuroendocrine markers. *Histopathology* **78**, 896-904 (2021).

7. Davis, A., Mahar, A., Wong, K., Barnet, M. & Kao, S. Prolonged Disease Control on Nivolumab for Primary Pulmon/ary NUT Carcinoma. *Clin Lung Cancer* **22**, e665-e667 (2021).

8. Patel, S.A.*, et al.* A #of metastatic NUT carcinoma with prolonged response on gemcitabine and n/ab-paclitaxel. *Clin #Rep* **9**, e04616 (2021).

9. Tosic, L.*, et al.* Intra-, para-, and suprasellar nuclear protein of testis carcinoma with infiltration of cavernous sinus and clivus-a #report. *Acta Neurochir (Wien)* **164**, 1105-1110 (2022).

10. Gupta, R.*, et al.* NUT midline lung cancer: a rare #report with literature review. *AME #Rep* **6**, 2 (2022).

11. Badran, A.*, et al.* Suspected NUT carcinoma progressing on pembrolizumab, carboplatin, and paclitaxel as first-line treatment: a #report. *Ann Med Surg (Lond)* **86**, 1061-1065 (2024).

12. Kloker, L.D.*, et al.* Clinical man/agement of NUT carcinoma (NC) in Germany: An/alysis of survival, therapy response, tumor markers and tumor genome sequencing in 35 adult patients. *Lung Cancer* **189**, 107496 (2024).

13. Matsuura, H.*, et al.* A Prompt Diagnosis and Treatment of a #of Nuclear Protein of the Testis Carcinoma Characterized by a Bronchial Lesion and High Serum Alpha-fetoprotein Level Following Genomic Testing. *Intern Med* **63**, 2655-2660 (2024).
